# The role of the *Alistipes* genus in intestinal inflammation, cancer, and aging: a narrative review

**DOI:** 10.3389/fmicb.2026.1822008

**Published:** 2026-05-21

**Authors:** Yan Zhang, Jinlong Xie, Xinyi Yan, Wenjie Zhao, Huimin Xu, Lilan Sun, Honggang Wang, Lu Zhao

**Affiliations:** 1 Center of Reproductive Medicine, Weifang People's Hospital, Shandong Second Medical University, Weifang, China; 2 Department of Medical Research Center, Weifang People's Hospital, Shandong Second Medical University, Weifang, China; 3 Department of General Surgery, Weifang People's Hospital, Shandong Second Medical University, Weifang, China; 4 Clinical Laboratory, Weifang People's Hospital, Shandong Second Medical University, Weifang, China

**Keywords:** aging, *Alistipes*, colorectal cancer, intestinal inflammation, prostate and bladder cancer

## Abstract

*Alistipes*, a bacterial genus under phylum Bacteroidetes, widely colonizes the gut. Since the first report of *Alistipes* in 2003, 13 species and 3 subspecies have been successfully isolated and identified from human feces, urine, appendiceal lesions, and rectal abscesses. *Alistipes* was reported to enhance the efficacy of immunotherapies in cancer treatment, while the detailed descriptions of other biological functions of this bacterium are lacking. This review summarizes the roles and potential mechanisms of *Alistipes* and its metabolites in inflammatory bowel diseases, digestive cancer, lung cancer, prostate and bladder cancer, and aging. Based on existing research, this narrative review provides a conceptual framework and emphasizes prospects for potential clinical value in diagnostics and therapeutics.

## 1 Introduction

Microorganisms are among the oldest forms of life on Earth, dating back to approximately 3.42 billion years (Cavalazzi et al., 2021). Since the emergence of humans, the human body has coexisted symbiotically with microorganisms, together forming a ‘superorganism,’ with the gut microbiota being the most abundant, and its total number nearly equal to that of human cells (Sender et al., 2016). Infant microorganisms are transmitted vertically from their mothers through vaginal delivery, breastfeeding, and other routes (de Vos et al., 2022; Browne et al., 2017). As people get older, the human microbiome plays a crucial role in host health.

*Alistipes* is an intestinal commensal genus belonging to the phylum Bacteroidetes. Members of this genus are gram-negative, slender, rod-shaped, strictly anaerobic bacteria that do not form spores and are inactivated by heating at 80 °C for 10 min (Bellali et al., 2019). Based on the NCBI Taxonomy Database (txid239759), 13 *Alistipes* species have been validly published, and most of them were discovered in clinical samples, including urine, feces, and abdominal abscesses. An increasing number of studies have confirmed that *Alistipes* and its metabolites are not only closely associated with various digestive system diseases, but also exert significant effects on extraintestinal cancers, including hepatoma, cholangiocarcinoma, pancreatic ductal adenocarcinoma, esophageal cancer, gastric cancer, lung cancer, prostate and bladder cancer (Cui et al., 2024; Mao et al., 2021; Dora et al., 2023; Matsushita et al., 2022; Zhu et al., 2025; Li et al., 2021). Besides, the abundance of *Alistipes* in the gut may be related to aging (Langille et al., 2014; Wilmanski et al., 2021; Luan et al., 2020). The mechanisms by which *Alistipes* contributes to the pathogenesis of multiple systemic diseases may act through the “gut–organ axis” (Lin X. et al., 2025). *Alistipes* plays diverse roles in different diseases, yet no existing review has comprehensively summarized recent advances regarding *Alistipes* species in human diseases. Accordingly, we summarize and integrate the dual or complex roles of *Alistipes* in disease progression, immunotherapy efficacy, and aging in this narrative review. The aims of this review are to elucidate the biological functions of *Alistipes* in various diseases and analyze its potential diagnostic and therapeutic value as biomarkers.

## 2 General characteristics of *Alistipes*

Colonies of *Alistipes* on blood agar are typically round, gray to opaque (translucent for *A. obesi*), with a diameter of 0.3–1.0 mm. All species are non-motile except for *A. obesi*. Some species, such as *A. onderdonkii* and *A. shahii*, produce non-fluorescent pigments on rabbit blood agar. Most *Alistipes* species are fermentative, producing putrefactive metabolites including ammonia, indole, phenols, histidine-derived compounds, and short-chain fatty acids (SCFAs). The detailed characteristics of 12 species are listed in Table 1, whereas those of *A. massiliensis* remain missing (Mailhe et al., 2016). We focus on the pathogenic roles of the *Alistipes* genus in human diseases, summarize available published literature, and outline the core biological functions of each *Alistipes* species in Table 2.

**Table 1:** Differential characteristics of the *Alistipes* strains.

Characteristic	1	2	3	4	5	6	7	8	9	10	11	12
References	Rautio et al. (2003)	Rautio et al. (2003)	Song et al. (2006)	Song et al. (2006)	Nagai et al. (2010)	Lagier et al. (2012)	Mishra et al. (2012)	Hugon et al. (2013)	Pfleiderer et al. (2014)	Shkoporov et al. (2015)	Bellali et al. (2019)	Bellali et al. (2019)
Cell diameter (μm)	0.2	0.4	0.35	0.15	0.6	0.62	0.56	0.6	0.72	0.56	0.5	0.45
G + C content (mol%)	57	55	58	56	55.2	58.8	58.4	58.6	57.9	56.6	58.6	58.3
Metabolism	F	NF	F	F	F	NF	F	NF	/	F	/	/
Oxygen requirement	−	−	−	−	−	−	−	−	−	−	−	−
Gram stain	−	−	−	−	−	−	−	−	−	−	−	−
Motility	−	−	−	−	−	−	−	+	−	−	−	−
Endospore formation	−	−	/	−	−	−	−	/	−	−	−	−
Pigment	+	−	+	+	+	+	+	+	−	+	/	/
Resistance to 20% bile	+	−	+	+	−	/	/	/	/	−	/	/
Catalase	−	+	−	−	+	+	+	+	−	−	−	+
Urease	/	−	−	−	−	−	/	−	−	−	−	−
Indole	+	+	+	+	−	+	+	−	−	+	/	/
SCFA Profile*	S, a, p	S, a, i, b	S, a, p	S, a, p	S.A, b	S	/	/	/	/	/	/
Enzyme activities^†^												
α-Galactosidase	+	−	+	+	+	+	+	+	/	−	−	−
β-Galactosidase	+	−	+	+	+	+	+	+	−	−	+	+
β-Glucosidase	−	−	−	+	+	/	/	−	+	−	−	+
α-Fucosidase	+	−	−	+	+	/	/	−	/	−	+	−
N-acetyl-β glucosamine	+	−	+	+	+	+	/	+	+	−	−	−
Source	Appendix	Feces	Abdominal abscess	Appendix	Feces	Feces	Feces	Feces	Feces	Feces	Feces	Feces

Strains: 1, *A. finegoldii* AHN 2437^T^; 2, *A. putredinis* ATCC 29800^T^; 3, *A. onderdonkii* WAL 8169^T^; 4, *A. shahii* WAL 8301^T^; 5, *A. indistinctus* YIT 12061^T^; 6, *A. timonensis* JC136^T^; 7, *A. senegalensis* JC50^T^; 8, *A. obesi* ph8^T^; 9, *A. ihumii* AP11^T^; 10, *A. inops* DSM 28863^T^; 11, *A. megaguti* P5997^T^; 12, *A. provencensis* P2431^T^. *a, Acetic acid; b, Butyric acid; i, Isobutyric acid; p, Propionic acid; s, Succinic acid. Capital letters denote major products and lowercase letters denote secondary products. ^†^Data were sourced from the API ZYM system. /, Data not found; F, Fermentative; NF, non-fermentative.

**Table 2:** The core biological functions of each *Alistipes* species in human diseases.

Species	Biological functions
*A. finegoldii*	1. Negatively correlated with IBD (de Meij et al., 2018; Malham et al., 2025). 2. Promotes pro-inflammatory cytokine expression in IBD under high-fat diet (Hou et al., 2022; Radka et al., 2022). 3. Enhances anti-PD-1 immunotherapy efficacy in solid tumors (Wu et al., 2025). 4. Positively correlates with PFS and durable clinical benefit in lung cancer patients (Dora et al., 2023). 5. Enriched in long-lived women (≥90 years) (Zhang et al., 2024).
*A. putredinis*	1. Negatively correlated with IBD (de Meij et al., 2018). 2. Active anti-inflammatory factor IL-10 via TLR2 to inhibit IBD (Ishikawa et al., 2024). 3. Reduces triglycerides, hepatic fat and inflammation in MASLD mice (Zhang et al., 2025). 4. Suppress LUAD via immunomodulation (Chen Z. et al., 2024). 5. Enriched in long-lived women (≥90 years) (Zhang et al., 2024).
*A. onderdonkii*	1. Biomarker for anastomotic healing and recurrence risk post CRC surgery (Hajjar et al., 2023). 2. Enhances anti-PD-1 immunotherapy efficacy in CRC (Xin et al., 2025). 3. Inhibits pancreatic cancer cell proliferation (Lee et al., 2021). 4. Positively correlates with PFS and durable clinical benefit in lung cancer patients (Haberman et al., 2023).
*A. shahii*	1. Ameliorates IBD (Lin X. et al., 2025). 2. Biomarker for anastomotic healing and recurrence risk post CRC surgery (Li et al., 2019). 3. Positively correlates with PFS and durable clinical benefit in lung cancer patients (Dora et al., 2023). 4. Modulates gut microbiome to generate lurasidone, suppressing BC (Zhu et al., 2025). 5. Enriched in long-lived women (≥90 years) (Zhang et al., 2024).
*A.indistinctus*	1. Induces IL-6 production to activate mast cell proliferation and elevate TNF-α levels, thereby accelerating CRC growth (Liu J. et al., 2023).
*A. timonensis*	1. Suppresses inflammation in colitis mice via OMVs and lipidome modulation (Older et al., 2025).
*A. senegalensis*	1. Ameliorates aging-related intestinal barrier dysfunction and systemic inflammation via indole synthesis, AhR activation, increased crypt length and goblet cell counts (Wang et al., 2025).
*A. megaguti*	1. Mediate bilirubin conversion via β-glucuronidase, inhibit intestinal PA and alleviate IBS symptoms (Edwinson et al., 2022).
*A. provencensis*	1. The same as *A. megaguti* (Edwinson et al., 2022).

## 3 Research on the role of *Alistipes* in intestinal inflammation

### 3.1 Research on the role of *Alistipes* in inflammatory bowel disease

Inflammatory bowel disease (IBD) is a chronic, recurrent inflammatory disorder of the intestines, encompassing ulcerative colitis (UC) and Crohn’s disease (CD). IBD compromises intestinal barrier function, mainly because excessive pathogenic gut microorganisms invade the mucosal lamina propria, leading to increased intestinal permeability (Christovich and Luo, 2022).

Clinical studies on the abundance of *Alistipes* in the gut microbiota with IBD showed that *A. finegoldii* and *A. putredinis* levels were significantly lower in IBD children than in healthy children (de Meij et al., 2018; Malham et al., 2025), and interestingly, the abundance of *Alistipes* was also reduced in pregnant women with IBD and their newborns (Kim et al., 2021). Moreover, the results of animal experiments showed that *A. finegoldii* was decreased in mice with UC (Dziarski et al., 2016), which was consistent with the results of clinical research. Above results suggested that the abundance of *Alistipes* in the gut microbiota was a potential biomarker for the diagnosis of IBD.

A large number of animal experiments investigated the effect of *Alistipes* spp. on inflammation and immune factors in IBD. For example, *A. putredinis* isolated from UC patients was transplanted to mice, and was found to promote anti-inflammatory interleukin (IL)-10 production via toll-like receptor 2 (Ishikawa et al., 2024). In NOD2-gene knockout mice, elevated *Alistipes* abundance reduces CD risk by modulating CD4+LAP+Foxp3− cells (Butera et al., 2018; Nayar et al., 2021). *A. onderdonkii* administration to mice significantly reduced tumor necrosis factor *α* (TNF-α) secretion in CD4+Foxp3−CD44hi and CD8+CD44hi T cells, thereby suppressing inflammatory responses (Li Z. et al., 2023). *A. timonensis* suppresses inflammation in colitis mice through outer membrane vesicles (OMVs) and lipidome modulation. These OMVs suppress inflammatory markers in plasma macrophages while affecting host plasma lipid levels (Toyofuku et al., 2023; Older et al., 2025). Oral administration of *A. shahii* upregulates tight junction proteins (ZO-1 and Claudin-1) and DDIT4 gene expression, inhibits mTOR and NLRP3 pathway activity, and promotes IL-10 release (Lin X. et al., 2025). In addition, the SCFAs produced by *Alistipes* spp. enhance intestinal barrier function and reduce inflammation: propionic acid promotes goblet cell differentiation and mucus gene expression, enhancing the intestinal mucus barrier in IBD mice (Wang X. et al., 2024); acetic acid directly protects the intestinal barrier integrity in patients with UC (Deleu et al., 2023); and butyric acid promotes M2 polarization of intestinal macrophages, restores goblet cell numbers in colitis mice (Liang et al., 2022; Gong et al., 2023).

Nevertheless, the functional role of *Alistipes* is not uniformly protective, and other evidence suggests potential pathogenic mechanisms under specific conditions. Increased levels of *Alistipes*, induced by enterobactin as an iron source, promote the release of proinflammatory cytokines through IL-6-mediated signal transducer and activator of transcription-3 (STAT3) signaling in lipocalin-2 (LCN2)/IL-10 double-knockout mice, inducing UC (Moschen et al., 2016). In studies of IBD drugs, the targets have been shown to inhibit LpxA and KdsB enzymes in lipid A synthesis, and the possible reason is disruption of the *Alistipes* cytoskeleton to alleviate symptoms (Sohail et al., 2025). OMVs secreted by *A. finegoldii* contain sulfonolipids produced following the administration of a high-fat diet, increasing inflammatory cytokine expression (Hou et al., 2022; Radka et al., 2022). Also, succinic acid produced by *Alistipes* is a pro-inflammatory metabolite that exacerbates the inflammatory response of macrophages, thereby aggravating intestinal inflammation (Fremder et al., 2021).

**Figure 1:**
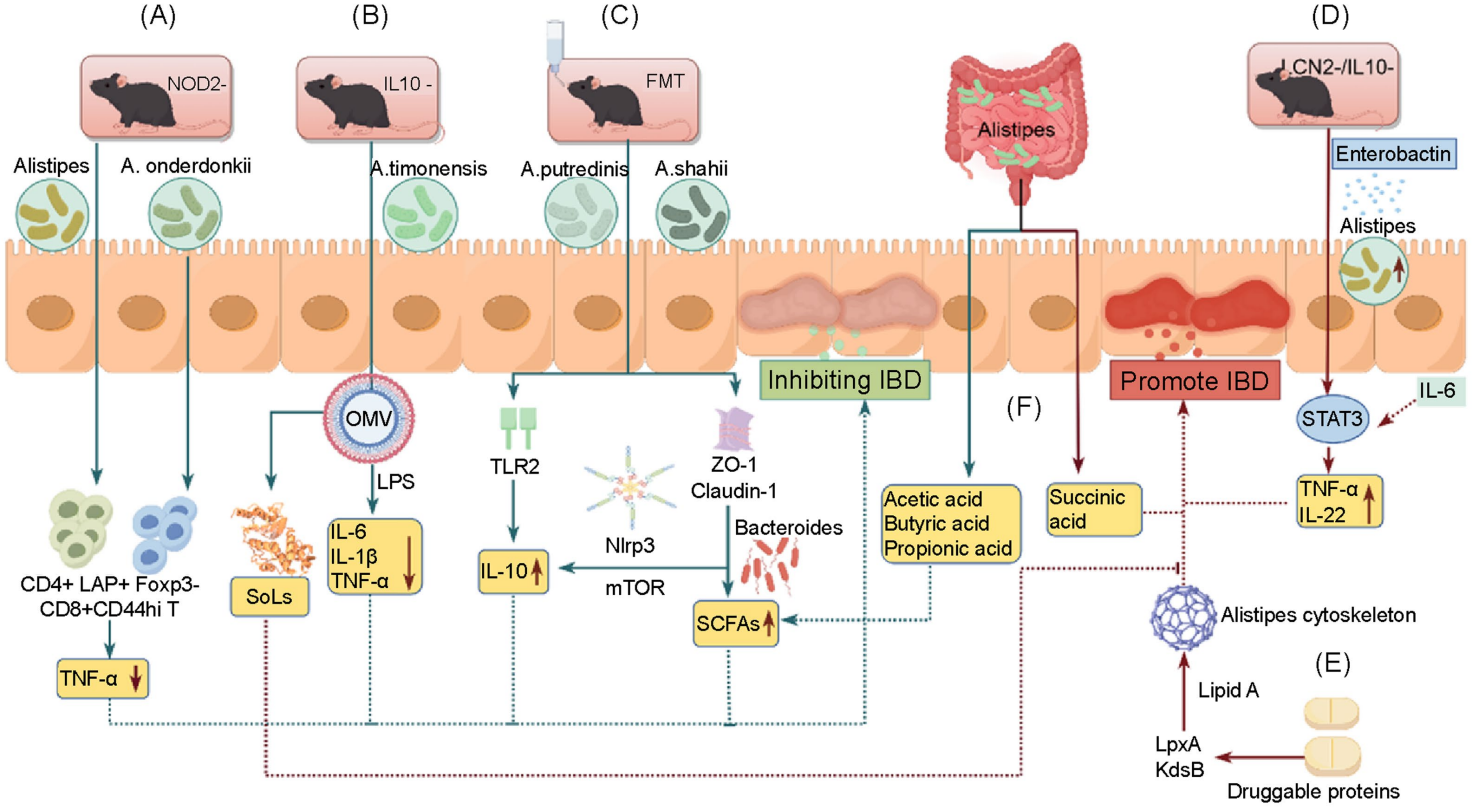
*Alistipes* participates in IBD pathogenesis. *Alistipes* contributes to IBD suppression: in NOD2 gene knockout mice, *Alistipes* and *A. onderdonkii* separately regulate the expression of CD8^+^CD44^hi^ and CD4^+^LAP^+^Foxp3^−^ cells, inhibiting TNF production **(A)**. In IL-10-deficient mice, *A. timonensis* suppresses LPS production and induces IL-6, IL-1*β*, and TNF-*α* expression via released OMVs **(B)**. *A. putredinis* and *A. shahii* induce IL-10 through TLR2 and tight junction protein promotion, respectively **(C)**. *Alistipes* contributes to IBD promotion: *Alistipes* uses enterobactin as an iron source, proliferates extensively in LCN2 and IL-10 double-knockout mice, activates IL-6-mediated STAT3 signaling, and promotes TNF-α and IL-22 release **(D)**. Druggable proteins disrupt *Alistipes* by inhibiting the key LPS A synthesis enzymes LpxA and KdsB and disrupting its cytoskeleton **(E)**. Succinic acid exacerbates inflammatory responses, whereas propionic acid, acetic acid, and butyric acid exhibit intestinal barrier protection and anti-inflammatory effects **(F)**. IBD, Inflammatory bowel disease; NOD2, nucleotide-binding oligomerization domain 2; OMV, outer membrane vesicle; TLR2, toll-like receptor 2; LCN2, lipocalin-2 (LCN2); IL, interleukin; STAT3, signal transducer and activator of transcription-3; TNF, tumor necrosis factor; LPS, lipopolysaccharide.

We present a schematic diagram to summarize the bidirectional effects of *Alistipes* on IBD in Figure 1, and analyze several reasons for this contradiction: (1) Changes in host genetic background: In LCN2/IL-10 double-knockout mice, this represents an extreme and specific immune condition, in which bacterial iron uptake and immune regulation are impaired, potentially altering the pathogenic potential of *Alistipes*. This is not applicable to all IBD patients, as in the NOD2-knockout model mentioned above, *Alistipes* shows protective effects (Butera et al., 2018; Nayar et al., 2021). (2) Strain-specific effects: *A. putredinis* and *A. shahii* primarily exert anti-inflammatory effects by inducing IL-10 production. However, most previous studies only identified *Alistipes* at the genus level due to limitations of 16S rRNA sequencing, and the functional characteristics of distinct species may differ substantially. (3) Abundance and stage-dependence: When *Alistipes* is present in low abundance, it may play a protective role in the early stage of disease by promoting the generation of regulatory T cells, whereas in established inflammation, overproliferation may promote inflammation through IL-6/STAT3 activation. (4) Dietary factors significantly influence the role of *Alistipes*. For instance, sulfonolipids in its OMVs exacerbate inflammation specifically under high-fat diet conditions (Hou et al., 2022; Radka et al., 2022), whereas a normal diet may favor its protective effects.

### 3.2 Research on *Alistipes* in irritable bowel syndrome and functional constipation

Irritable bowel syndrome (IBS) is a chronic intestinal disorder characterized by abdominal pain and bowel dysfunction, in the absence of significant organic lesions (Black and Ford, 2020). Elevated proteolytic activity (PA) has been detected in stool supernatants and colonic mucosa of patients with IBS, which may contribute to intestinal barrier dysfunction and visceral hypersensitivity (Hou et al., 2021). *A. megaguti* and *A. provencensis* mediate bilirubin conversion via *β*-glucuronidase, thereby inhibiting intestinal PA and alleviating IBS symptoms (Edwinson et al., 2022). The relative abundance of *Alistipes* is reduced in IBS patients compared with healthy controls (Chen J. et al., 2024), and fecal microbiota transplantation (FMT) significantly ameliorates IBS symptoms while increasing fecal *Alistipes* levels (El-Salhy, 2023). Therefore, *Alistipes* may benefit patients with IBS and offer novel therapeutic strategies for IBD.

Fecal constipation (FC) is a type of constipation without organic pathology, characterized by straining during bowel movements, reduced frequency, and incomplete evacuation. Gut microbiota dysbiosis is instrumental in FC development, and restoring microbial balance alleviates the symptoms (Sperber et al., 2021). Patients with FC have a significantly higher abundance of the genus *Alistipes* in gut microbiota compared with healthy controls (Hu et al., 2025; Sugitani et al., 2021), and this phenomenon has also been confirmed in the pediatric population (Zhao et al., 2025). Based on this microbial community characteristic, we speculate that it may be involved in the pathogenesis of FC by interfering with tryptophan metabolism: existing studies have revealed that tryptophan metabolism mainly includes two key pathways—the gut microbiota-mediated indole metabolism pathway and the host 5-hydroxytryptamine (5-HT) synthesis pathway (Bai et al., 2022). The increased abundance of *Alistipes* may enhance its efficiency in converting dietary tryptophan into indole and its derivatives, thereby competitively inhibiting the biosynthesis of 5-HT in enterochromaffin cells. As a key neurotransmitter regulating gut motility, decreased 5-HT levels can prolong the transit time of feces in the colon through the gut-brain axis signaling pathway, thus contributing to the occurrence and development of FC (Bai et al., 2022; Chang et al., 2023). However, these reported results only demonstrate an association between 5-HT and *Alistipes* in FC; more direct evidence is needed to establish causality. Future studies should address: (1) the precise effects of specific *Alistipes* species; (2) the direct causal relationship between indole metabolic dynamics and host 5-HT regulation; and (3) validation through isotope tracing and germ-free animal colonization experiments.

## 4 Research on the role of *Alistipes* in cancer

### 4.1 Research on the role of *Alistipes* in CRC

Colorectal cancer (CRC) ranks third globally in incidence and fourth in mortality among all cancers (Tito et al., 2024). Core gut microbiota serves as indicators and therapeutic targets for CRC (Janney et al., 2020; Tito et al., 2024; Lin Y. et al., 2025). Metagenomic sequencing showed higher levels of *Alistipes* in tumor tissues in advanced-stage CRC patients compared with those of early-stage patients and healthy controls (Hasan et al., 2022). As CRC progresses, the number of OMVs secreted by *Alistipes* significantly increased in the gut of patients (Park et al., 2021). In the “adenoma-carcinoma sequence” model, *Alistipes* shows persistent enrichment as cells progress from adenoma to carcinoma (Sun et al., 2017; Jing et al., 2024). Specific *Alistipes* species, such as *A. shahii* and *A. onderdonkii*, can serve as biomarkers to predict anastomotic healing and recurrence risk following CRC surgery (Li et al., 2019; Hajjar et al., 2023). These associations suggest that *Alistipes* may play a potential promoting role in the progression of CRC, but the causal relationship between *Alistipes* and the disease requires further experimental validation.

**Figure 2:**
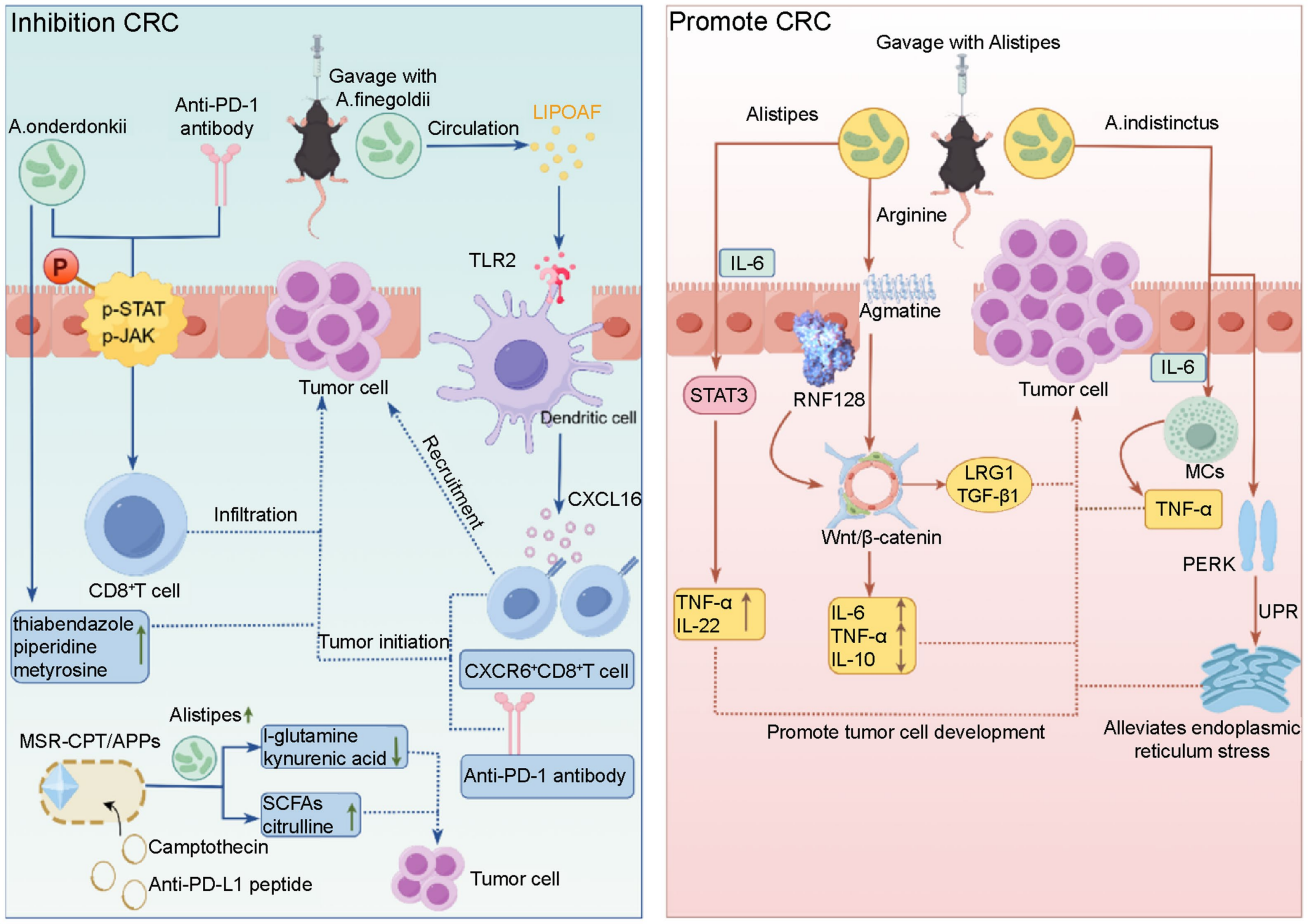
*Alistipes* involvement in the pathogenesis of colorectal cancer. Left panel: *A. onderdonkii* combined with anti-PD-1 therapy promotes CD8^+^ T cell infiltration into the tumor microenvironment and increases the levels of tumor-suppressive metabolites (thiabendazole, piperidine, and D/L-α-metyrosine). *A. finegoldii* enhances CXCR6^+^ CD8^+^ T cell recruitment to the tumor microenvironment, amplifies anti-PD-1 immunotherapy efficacy, and inhibits CRC development. Oral administration of MSR-CPT/APPs loaded with camptothecin and anti-PD-L1 peptides remodels the tumor immune microenvironment, alleviates immunosuppression, and effectively eradicates primary and metastatic colorectal tumors. Right panel: *Alistipes* activates the STAT3 pathway via IL-6, releasing proinflammatory factors such as TNF-α and IL-22. *Alistipes* uses arginine to generate guanidine butyric acid, which binds to E3 ubiquitin ligase RNF128, inhibits anti-inflammatory factors such as IL-10, and activates the LRG1/TGF-β1 pathway, leading to intestinal cell dysplasia and lymphocyte infiltration. *A. indistinctus* exacerbates CRC progression by inducing IL-6 production, upregulating PERK expression, and activating downstream UPR. CRC, colorectal cancer; MSR-CPT/APPs, oncolytic magnetotactic bacteria loaded with camptothecin and anti-PD-L1 peptides; STAT3, signal transducer and activator of transcription-3; IL, interleukin; TNF, tumor necrosis factor; LRG1, leucine-rich ɑ2-glycoprotein-1; TGF-β1, transforming growth factor-β1; UPR, unfolded protein response.

Based on the published studies, *Alistipes* may promote CRC through three main pathways (Figure 2). First, *Alistipes* can degrade arginine into agmatine (Lu et al., 2024; Yu et al., 2024), and agmatine can bind to the E3 ubiquitin ligase RNF128, thereby activating the Wnt/*β*-catenin signaling pathway in CRC cell models and this pathway activation increases the release of inflammatory factors (such as IL-6 and TNF-*α*) and is accompanied by upregulation of the LRG1/TGF-β1 pathway, which promotes CRC progression (Fu et al., 2024). However, the causal effect of the above proposed mechanism *in vivo* still needs further verification. For example, studies are needed to confirm the interaction between RNF128 and agmatine in human CRC tissues and to determine whether *Alistipes* directly activates the Wnt signaling pathway in cellular or mouse models to establish a clear causal relationship. Second, *A. indistinctus* induces production of IL-6, which activates mast cell proliferation, increasing TNF-α to accelerate tumor cell growth (Desai et al., 2016; Dal Secco et al., 2025; Liu J. et al., 2023). The bacterium can also activate protein kinase R-like endoplasmic reticulum kinase (PERK) and unfolded protein response (UPR), while cancer cells utilize the UPR to survive adverse conditions and accelerate drug resistance (Liu J. et al., 2023). Third, the increase in IL-6 caused by *Alistipes* also activates the STAT3 signaling pathway, which could inhibit apoptosis, promote glycolysis, induce epithelial-mesenchymal transition (EMT), and drive tumor metastasis (Hashemi et al., 2023; Sun et al., 2023), and STAT3 induces the release of TNF-α and IL-22 leading to the progression of CRC (Moschen et al., 2016).

However, results from other studies indicate that *Alistipes* plays a role in antitumor activity by modulating immune responses and alleviating intestinal inflammation in CRC (Wang et al., 2021; Shakhpazyan et al., 2024). Combined treatment with *A. onderdonkii* and anti-PD-1 antibody can reduce the expression of p-JAK and p-STAT, downregulate PD-L1, and enhance CD8+ T cell infiltration into the tumor microenvironment. Meanwhile, this combination also elevates levels of tumor-suppressive metabolites such as thiabendazole and piperidine (Xin et al., 2025). Other researchers have found that *A. finegoldii* combined with PD-1 therapy inhibits tumor progression via lipoprotein LIPOAF-mediated activation of the NF-κB pathway, which induces CXCL16 expression in CCR7+ dendritic cells and promotes CXCR6+CD8+ T cell enrichment (Wu et al., 2025). The above research suggests that PD-1 inhibitors, a widely used anti-cancer drug, when used in combination with *Alistipes*, can significantly enhance the anti-CRC effect. MSR-CPT/APPs for Oral administration increases the abundance of *Alistipes* and its beneficial metabolites (SCFAs and citrulline), reduce harmful substances (L-glutamine and kynurenic acid), and eliminates colorectal tumors (Jiang et al., 2025). Notably, SCFAs as bacterial metabolites can reduce the risk of CRC through epigenetic mechanisms including DNA methylation, histone modifications, and non-coding RNAs (Thulasinathan et al., 2025). The above findings are summarized in Figure 2.

Regarding the two contradictory results of the role of *Alistipes*, we considered the reasons as follows: (1) Functional differences among species: for example, *A. finegoldii*, *A. onderdonkii*, and *A. shahii* have distinctly different metabolic and immune-regulatory characteristics, so the reactions caused in CRC are different; (2)Different gut microenvironment and inflammatory states may determine the final phenotype of bacteria; (3) Diet and environmental factors intricately regulate gut microbial homeostasis and *Alistipes* function, rendering simple pro- or anti-cancer classification overly simplistic. Future studies should integrate multi-omics, strain functional validation, and gnotobiotic animal models to systematically establish causality and underlying mechanisms between *Alistipes*, gut microbial balance, and CRC.

### 4.2 Research on the role of *Alistipes* in hepatobiliary and pancreatic diseases

The concept of the “gut–liver axis,” proposed by Marshall, has established “gut-based therapy for liver diseases” as a significant research direction (Pabst et al., 2023). Regarding liver diseases, metagenomic analysis has revealed the presence of microorganisms in tumor and adjacent non-tumor tissues of pediatric patients with hepatoblastoma (HB), with the abundance of *Alistipes* being significantly lower in tumor tissues (Cui et al., 2024), indicating that *Alistipes* may be negatively correlated with HB. In a mouse model of metabolic dysfunction-associated steatotic liver disease (MASLD), administration of *A. putredinis* reduced serum triglyceride levels and hepatic steatosis, inhibited weight gain, and alleviated pathological features of MASLD, while decreasing inflammatory factors and increasing the abundance of beneficial bacteria including Lachnospiraceae and *Akkermansia* (Zhang et al., 2025). It has also been found in a clinical study that *Alistipes* abundance is higher in healthy individuals than in MASLD patients, and that *Alistipes* levels are negatively correlated with serum glucose, gamma-glutamyltransferase, and alanine aminotransferase (Jiang et al., 2015; Yang et al., 2023). These research results suggest that *Alistipes* may be associated with liver function, but whether or not it can serve as a potential biomarker for liver function still needs to be further studied in larger sample cohorts.

Cholangiocarcinoma (CCA) is a malignant tumor originating from the epithelial cells of the biliary tract (Wilechansky, 2025). Ultrasound or CT imaging has limited specificity and sensitivity, making it difficult to detect early-stage CCA when symptoms are hidden (Silsirivanit et al., 2020). To date, there are no specific non-invasive biomarkers for CCA screening. Increasing evidence indicates that specific gut microbiota composition is closely associated with CCA development and may influence the patient’s immunotherapy efficacy. Zhang et al. found that the abundance of *Alistipes* in the stool was significantly higher in CCA patients than in those with cholelithiasis and healthy individuals (Zhang et al., 2021). The abundance of *Alistipes* is positively correlated with risk of CCA through regulating the AMPK and mTOR signaling pathways to promote tumorigenesis (Chen et al., 2023). Surprisingly, hepatobiliary cancer patients receiving PD-1/PD-L1 inhibitor therapy, the higher abundance of *Alistipes*, the better clinical benefit response, the longer progression-free survival (PFS) and overall survival (OS), and the researchers also found the *Alistipes* positively correlates with metabolites enriched in the durable clinical benefit patients (e.g., 4-[(hydroxymethyl) nitrosoamino]-1-(3-pyridyl)-1-butanone, NNK), while exhibiting negative correlations with metabolites enriched in the non-durable clinical benefit patients (e.g., pyrrolidine) (Mao et al., 2021; Zhu et al., 2024). These findings indicate that Alistipes may be associated with cholangiocarcinoma and have the potential to serve as a non-invasive diagnostic indicator. It may also help predict the efficacy of immunotherapy for cholangiocarcinoma. However, its clinical application is limited due to the small sample size and unclear causal relationship, requiring further large-sample studies to confirm its practical value.

Pancreatic ductal adenocarcinoma (PDAC) is a highly malignant tumor characterized by poor prognosis and high mortality (Yu et al., 2025). An *in vitro* study showed that co-culture of primary pancreatic cancer cells with *A. onderdonkii* inhibited cancer cell proliferation. Transcriptomic analysis further identified altered expression of genes associated with cell proliferation and apoptosis, including *ANKRD37*, *PPP1R3C*, *ENO2*, *NSG1*, and *CABLES1* (Lee et al., 2021). Furthermore, the abundance of *A. onderdonkii* was significantly reduced in PDX mice that received FMT from PDAC patients (Lee et al., 2021). Similarly, its abundance was significantly lower in the intestines of patients with advanced unresectable PDAC than in those with early-stage resectable disease (Guo et al., 2022). These preliminary findings suggest a potential association between *Alistipes* abundance and PDAC progression, although further mechanistic studies and clinical validation are required to clarify its exact role in disease development.

### 4.3 Research on the role of *Alistipes* in esophageal and gastric cancers

The primary subtype of esophageal cancer (EC) is esophageal squamous cell carcinoma (ESCC) (Cammarota et al., 2025). Owing to insidious early symptoms of ESCC, most patients are diagnosed as having advanced stage disease for the first time, so it is critical to develop non-invasive, highly sensitive, and specific biomarkers for early diagnosis and screening. Studies have shown significant differences in gut metabolomic profiles between patients with ESCC and healthy controls, with indoles and their derivatives—key differential metabolites—being significantly elevated in ESCC patients. Spearman correlation analysis indicates that the genus *Alistipes* may be involved in the metabolism of indoles and their derivatives, and is closely associated with the occurrence and development of ESCC (Gao et al., 2024). However, inconsistencies remain in current research regarding the role of *Alistipes* in EC: a Mendelian randomization analysis indicated that high *Alistipes* abundance may increase the risk of Barrett’s Esophagus (BE) (Li et al., 2024), while another study suggested that *Alistipes* may exert an inhibitory effect on the progression of BE and esophageal adenocarcinoma (EAC) (Yang et al., 2022). This discrepancy may stem from fundamental differences in tissue origin and molecular mechanisms between BE—a well-established precancerous lesion of EAC—and ESCC (Deboever et al., 2024). Thus, the functional heterogeneity of *Alistipes* across different esophageal tumor subtypes still requires further verification through animal models and in vitro experiments.

Previous studies have confirmed that the abundance of *Alistipes* in the feces of patients with gastric cancer (GC) is significantly higher than that in healthy individuals (Li et al., 2021). However, a recent study demonstrated that *Alistipes* abundance is inversely correlated with GC risk, which may be attributed to its modulation of the side scatter area of natural killer T (NKT) cells, thereby reducing the incidence of GC (Wang C. et al., 2024). As key lymphocytes in the innate immune system, NKT cells play a crucial role in inhibiting the occurrence, development, and metastasis of GC (Sun et al., 2024). Nevertheless, these findings show obvious inconsistencies regarding the association between *Alistipes* and GC risk. Current studies are limited by small sample sizes, cross-sectional designs, and insufficient mechanistic evidence, which may partly explain these conflicting results.

### 4.4 Research on the role of *Alistipes* in lung cancer

Numerous studies have demonstrated that gut microbiota modulates lung cancer initiation and progression via the gut–lung axis, a bidirectional communication network between the gut and lungs (Budden et al., 2017; Ankudavicius et al., 2024; Song et al., 2024). Specifically, the gut microbiota and its metabolites can traverse the intestinal epithelial barrier to enter the systemic circulation, thereby regulating pulmonary inflammation by promoting the differentiation of regulatory T cells (Ashique et al., 2022; Budden et al., 2017). Conversely, pulmonary infections can trigger systemic immune responses, leading to the release of inflammatory mediators and stress hormones that disrupt the gut microbiota and induce intestinal damage (Song et al., 2024). Clinical investigations have revealed that fecal *Alistipes* abundance is significantly elevated in patients with non-small cell lung cancer compared with healthy controls (Ankudavicius et al., 2024). Moreover, *A. shahii*, *A. finegoldii*, and *A. onderdonkii* are positively correlated with PFS and enhanced durable clinical benefit in patients with lung cancer (Dora et al., 2023; Haberman et al., 2023). A Mendelian randomization analysis indicated that *A. putredinis* may inhibit lung adenocarcinoma (LUAD) through immune regulation. Specifically, univariate and multivariable MR mediation analyses identified CCR7 on naive CD8 + T cells as a significant mediator, with a mediation proportion of 9.5% (*p* = 0.018), which suggested *A. putredinis* modulates CD8 + T cell function via chemokine receptor signaling to exert protective effects against lung cancer (Chen J. et al., 2024; Chen Z. et al., 2024). For patients undergoing lung cancer surgery, *Alistipes* enhanced postoperative outcomes by its metabolism SCFAs, such as butyric acid, thereby enhancing specific airway conductance and exercise tolerance (Marfil-Sanchez et al., 2021). Collectively, *Alistipes* may exert beneficial effects in patients with lung cancer. Nevertheless, these findings should be interpreted cautiously, as most evidence is limited by small sample sizes, inadequate adjustment for confounders including diet and medication, and insufficient mechanistic validation in retrospective studies. Although Mendelian randomization analysis implies potential causal relationships, it cannot confirm detailed biological pathways, which warrant further experimental verification.

### 4.5 Research on the role of *Alistipes* in prostate and bladder cancers

Prostate cancer (PCa) is the second leading cause of cancer-related deaths in men, following lung cancer (Matsushita et al., 2022; Liu Y. et al., 2023). FMT from castration-resistant prostate cancer (CRPC) patients into TRAMP mice resulted in the enrichment of broad-spectrum SCFA-producing bacteria, including *Alistipes*, and both SCFAs and *Alistipes* were shown to induce tumor cell autophagy and M2 macrophage polarization, thereby promoting PCa progression via the IGF-1 axis in non-specific metabolic pathways (Liu Y. et al., 2023; Matsushita et al., 2022). In summary, current research mainly regards *Alistipes* as a potential biomarker associated with PCa risk. Its pro-tumorigenic role remains speculative, and further studies using *Alistipes* mono-colonization in germ-free animal models are urgently needed to verify the causal relationship between *Alistipes* and PCa.

Statistically, the incidence and mortality rates of bladder cancer (BC) are higher in men than in women (Sung et al., 2021; Abdel-Hafiz et al., 2023; Zhu et al., 2025). Recent evidence indicates that *A. shahii* is significantly enriched in females, regardless of whether they are healthy individuals or BC patients (Zhu et al., 2025). Mechanistically, *A. shahii* acts in concert with the host gut microbiota to produce the metabolite lurasidone, which targets the iron-sequestering protein LCN2 to release Fe2 + and induce ferroptosis in RETNLG+LCN2 + senescence-like neutrophils (RLSN) (Note: This lurasidone is a microbial metabolite produced by bacteria, distinct from the synthetic pharmaceutical lurasidone.) This process is more pronounced in females due to higher levels of *A. shahii* and lurasidone, thereby eliminating immunosuppressive RLSN. Conversely, elevated systemic LCN2 in males suppresses the growth of iron-sensitive *Alistipes* species, establishing a feedback loop that promotes RLSN accumulation and tumor progression. Consistent with these results, clinical data have demonstrated higher RLSN infiltration and serum LCN2 in male BC patients, whereas female patients show elevated serum lurasidone; both parameters are correlated with prognosis (Zhu et al., 2025). Collectively, these findings suggest that the *Alistipes*-lurasidone-LCN2 axis may contribute to sexual dimorphism in BC development. However, lurasidone synthesis was undetectable in pure *A. shahii* cultures, suggesting that this process may involve multispecies co-metabolism or host–microbe interactions. Moving forward, lurasidone represents a promising therapeutic agent, since its administration was shown to abrogate sex-based disparities and enhance anti-PD-1 efficacy in murine models (Zhu et al., 2025).

**Figure 3:**
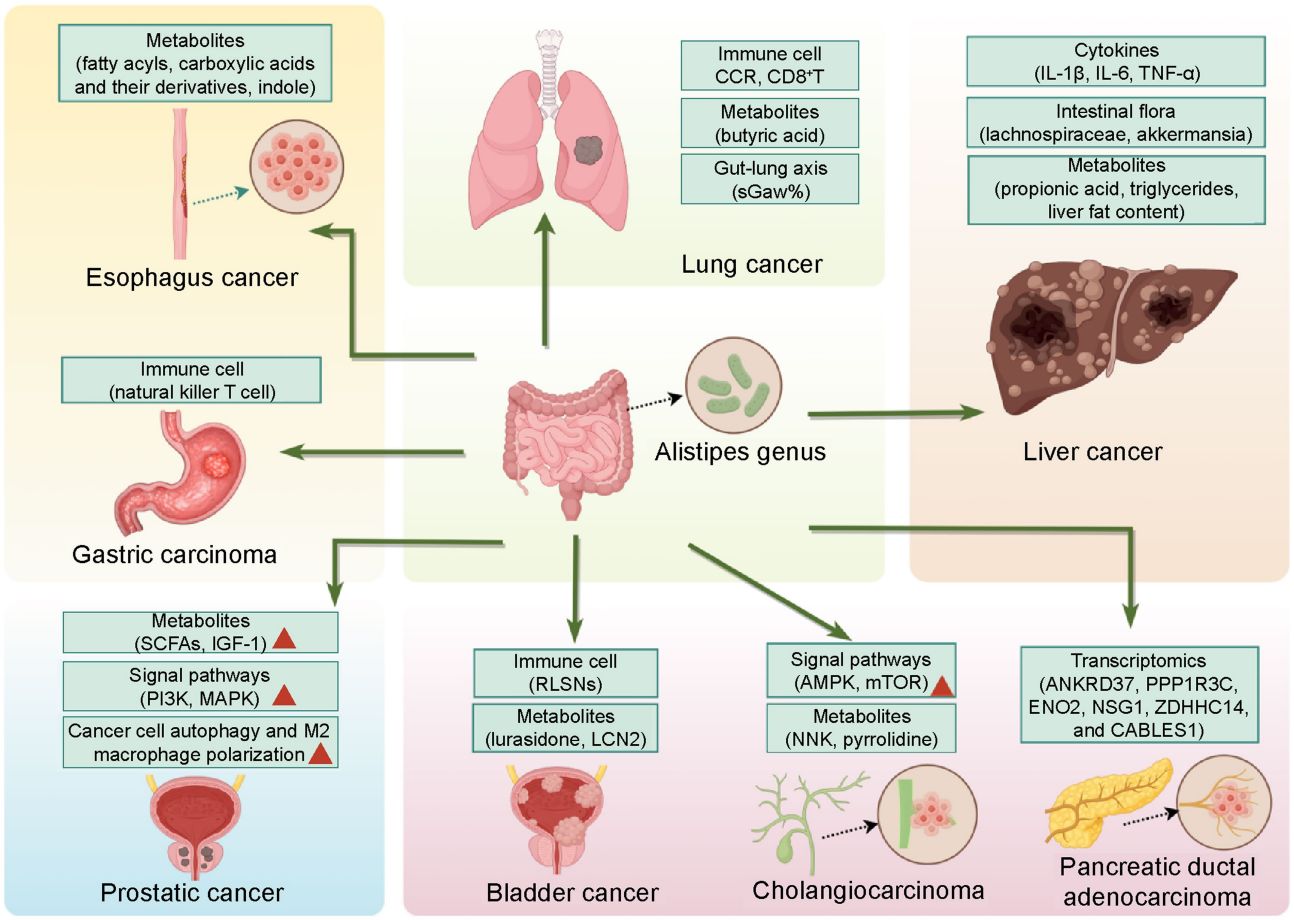
Hallmarks of the influence of the gut microbial genus *Alistipes* on various cancers. *Alistipes* influences human health through multiple pathways, including immune regulation, endocrine regulation, transcriptomic changes, microbial metabolites, the maintenance of intestinal microbiota balance, and interactions between the intestinal microbiota and organ axes. Effects or mechanisms related to the inhibition of tumor responses are listed with no symbols, whereas those related to a pro-tumor state are marked with a red triangle.

Finally, we summarize the key molecules and metabolites potentially modulated by *Alistipes* in cancers other than CRC in Figure 3, and highlight promising targets for the precision treatment of malignancies.

## 5 Research on the role of *Alistipes* in biological aging

Aging is a complex phenomenon accompanied by profound gut microbiome remodeling, characterized by three main features: decreased microbial diversity (Ghosh et al., 2022), expansion of pro-inflammatory taxa (Hohman and Osborne, 2022), and diminished metabolic capacity (Wang T. et al., 2024). Chronic inflammation, a hallmark of aging, impairs quality of life and drives immune cell senescence, compromising clearance of senescent cells and inflammatory mediators. This establishes a vicious “inflammation-aging” cycle that accelerates age-related decline and shortens lifespan (Hohman and Osborne, 2022; Li X. et al., 2023). Recent work demonstrates that *A. senegalensis* ameliorates age-related intestinal barrier dysfunction and systemic inflammation in mice by enhancing indole synthesis, activating the aryl hydrocarbon receptor, and increasing crypt length and goblet cell numbers (Wang et al., 2025). These findings suggest a potential role for *A. senegalensis* in supporting markers of intestinal health during aging. Consistently, *Alistipes* abundance is significantly elevated in individuals ≥65 years versus young controls, with antibiotic use further amplifying this enrichment (Wilmanski et al., 2021). In geriatric microbiomes, Bacteroidetes accounts for over half (53%) of core taxa—dominated by *Bacteroides* (29%) and *Alistipes* (17%)—compared with just 8–27% in young adults (Claesson et al., 2011). Similar age-associated enrichment of *Alistipes* is observed in aged mice (Langille et al., 2014; Wilmanski et al., 2021), indicating cross-species consistency. However, these associations remain correlative and potentially confounded by diet, medication, and other factors; causal relationships and underlying mechanisms require rigorous experimental validation.

Enrichment of *Alistipes* in centenarians and their relatives has been hypothesized to promote 3-oxoalloLCA synthesis, maintaining microbiome homeostasis and potentially extending lifespan (Sato et al., 2021). However, this remains speculative due to confounders inherent to centenarian populations. Age-associated decline in fecal SCFAs, particularly butyrate, coincides with reduced abundance of primary producers (*Faecalibacterium prausnitzii*, *Eubacterium rectale*) and compensatory expansion of *Alistipes* species (*A. shahii*, *A. onderdonkii*, *A. senegalensis*) that generate butyrate via the L-lysine pathway (Sato et al., 2021; Ravikrishnan et al., 2024). In germ-free mice, both exogenous butyrate supplementation and fecal transplantation from aged donors extend lifespan, possibly via upregulation of the longevity hormone FGF21 (Kundu et al., 2019). In summary, current evidence reveals a correlative association between *Alistipes* enrichment, altered butyrate metabolism, and longevity, but causal validation in humans is still lacking.

Notably, global female life expectancy (74 years) exceeds male (68 years), with women comprising most long-lived populations. The *Alistipes* genus can be vertically transmitted across three to four generations (Valles-Colomer et al., 2022), and in long-lived women (≥90 years), *A. finegoldii*, *A. putredinis*, and *A. shahii* are enriched 3.2-fold versus younger geriatric women (60–90 years) (Zhang et al., 2024). Furthermore, *A. finegoldii* and *A. shahii* decline precipitously 7 months before death in centenarians, suggesting these shifts may herald health deterioration (Luan et al., 2020). Collectively, *Alistipes* is associated with aging and female longevity, with consistent age-related enrichment observed. However, these associations are correlative rather than causal and may be confounded by diet, medication, or antibiotic use. Taken together, *Alistipes* may serve as a biomarker of aging and longevity, but current evidence is insufficient to confirm that it actively promotes healthy aging or lifespan. Further experimental validation is required to establish causality.

## 6 General conclusion and perspectives

*Alistipes* plays a significant role in host health, lifespan, and various diseases, influencing the progression of disorders related to the digestive, respiratory, urinary, nervous, and immune systems via the gut–organ axis. The dual role of *Alistipes* as a protective symbiont or a pro-inflammatory pathogen is not inherent; its metabolites are a key factor in gut inflammatory states, such as the balance between succinic acid and protective SCFAs. Disruption of this balance by external factors (e.g., antibiotics or immune dysregulation) may shift the functional phenotype of *Alistipes* toward pathogenicity. Thus, *Alistipes* metabolites represent a valuable research direction.

Most studies indicate that *Alistipes* is associated with health benefits and may synergize with immunotherapies (e.g., PD-1 inhibitors) to inhibit cancer progression. However, its role in CRC and IBD remains controversial, potentially due to the distinct characteristics of its 13 strains, its coexistence with other microorganisms, and environmental factors. Nevertheless, no single *Alistipes*-colonized animal models are available for validation, highlighting the need to integrate multi-omics and animal studies to distinguish correlation from causation.

Notably, existing evidence supports the potential of *Alistipes* as a diagnostic biomarker, prognostic indicator, and therapeutic target. Future research should incorporate preclinical models to explore the biological functions of this genus and its causal relationships with other pathogenic bacteria. Urgently needed is the integration of multi-dimensional approaches, including metagenomic sequencing (species/strain-level resolution), strain isolation and cultivation (functional validation), and single-strain colonization animal models (causal analysis), to systematically elucidate the causal links between *Alistipes* and diseases. This may enable targeted prevention and treatment of *Alistipes*-related diseases through interventions such as probiotic preparations and transplantation, thereby improving human health and longevity.

## Glossary

AMPK: AMP-activated protein kinase
BC: Bladder cancer
CCA: Cholangiocarcinoma
CCR7: C-C motif chemokine receptor 7
CD: Crohn’s disease
CRC: Colorectal cancer
CXCL16: C-X-C motif chemokine ligand 16
CXCR6: C-X-C motif chemokine receptor 6
EC: Esophageal cancer
ESCC: Esophageal squamous cell carcinoma
FC: Fecal constipation
FMT: Fecal microbiota transplantation
Foxp3: Forkhead box protein P3
GC: Gastric cancer
HB: Hepatoblastoma
HDAC3: Histone deacetylase 3
IBD: Inflammatory bowel disease
IGF-1: Insulin-like growth factor 1
JAK2: Janus kinase 2
LAP: Leucine aminopeptidase
LCN2: Lipocalin-2
LPS: Lipopolysaccharide
LRG1: Leucine-rich ɑ2-glycoprotein-1
LW: Long-lived women
MASLD: Metabolic dysfunction-associated steatotic liver disease
MC: Mast cell
mTOR: Mammalian target of rapamycin
NKT: Natural killer T
NOD2: Nucleotide-binding oligomerization domain 2
OMV: Outer membrane vesicle
OS: Overall survival
PA: Proteolytic activity
PCa: Prostate cancer
PD-1: Programmed cell death
PDAC: Pancreatic ductal adenocarcinoma
PDX: Patient derived xenograft
PERK: Protein kinase R-like endoplasmic reticulum kinase
PFS: Progression-free survival
RLSN: RETNLG+LCN2+aging-like neutrophil
SCFA: Short-chain fatty acid
STAT3: Signal transducer and activator of transcription-3
TGF-*β*: Transforming growth factor-β
TNF: Tumor necrosis factor
UC: Ulcerative colitis
UPR: Unfolded protein response
ZO-1: Zonula occludens-1
